# Intrahepatic intraductal papillary cystic neoplasm of the bile duct: A case report

**DOI:** 10.1016/j.amsu.2021.02.013

**Published:** 2021-02-12

**Authors:** Evangelos G. Baltagiannis, Christina Kalyvioti, Anastasia Glantzouni, Anna Batistatou, Petros Tzimas, Georgios K. Glantzounis

**Affiliations:** a HPB Unit, Department of Surgery, University Hospital of Ioannina and School of Medicine, University of Ioannina, Ioannina, Greece; b Department of Radiology, “G. Hatzikosta” General Hospital, Ioannina, Greece; c Department of Pathology, University Hospital of Ioannina and School of Medicine, University of Ioannina, Ioannina, Greece; d Department of Anesthesiology, University Hospital of Ioannina and School of Medicine, University of Ioannina, Ioannina, Greece

**Keywords:** Intraductal papillary neoplasm of the bile duct, Diagnosis, Management, Liver resection, Cystic liver lesion, Case report

## Abstract

**Introduction and importance:**

Intraductal papillary neoplasm of the bile duct (IPNB) is a tumour with a very low incidence in the Western world, characterised by a high risk of malignant transformation and unknown prognosis. It is a new entity which was adopted by the WHO in 2010 as a precursor lesion of cholangiocarcinoma. Intrahepatic bile duct is the most common site of origin for IPNB.

**Case presentation:**

Hereby, we present a case of an asymptomatic 63- year-old man, referred to our department after routine ultrasonography showing a multifocal cystic lesion on the left hepatic lobe. Further screening modalities (CT, MRI abdo) confirmed a complex cystic liver lesion with atypical features. The patient underwent left hepatectomy. Histopathology showed a cystic type intrahepatic IPNB, which was completely resected (R0). The follow up in 2 yrs post-operation showed no signs of recurrence.

**Clinical discussion:**

The diagnosis and management of IPNB remain challenging. A multimodality imaging approach is essential in order to diagnose IPNB, assess tumour location and extent and plan the optimal treatment strategy.

**Conclusion:**

Complete surgical resection (R0) with close postoperative follow-up offers long-term survival.

## Highlights

• Intraductal papillary neoplasm of the bile duct (IPNB) is a rare bile duct epithelial tumour with very low incidence in the West and constitutes a precursor to the development of cholangiocarcinoma.
• The diagnosis and management of IPNB remains challenging. A multimodality imaging approach is essential in order to diagnose IPNB, assess tumor location and extent and plan the optimal treatment strategy.
• Complete surgical resection (R0) with close postoperative follow-up offers long-term survival.

## 1 Introduction

Intraductal papillary neoplasm of the bile duct (IPNB) is a rare bile duct tumour which lacks ovarian type stroma, characterised by exophytic proliferation of biliary epithelium on fibrovascular stalks within the bile duct [1]. IPNB grows mainly within bile ducts (intrahepatic and extrahepatic), where gradually transforms from adenoma to adenocarcinoma and may invade the liver parenchyma surrounding the bile ducts in advanced stages [2]. An invasive component is present in approximately 40%–80% of reported cases. IPNBs are considered the biliary counterpart of pancreatic intraductal papillary mucinous neoplasms (IPMNs) [3]. As a new entry with an incredibly low incidence in the Western countries and incomplete understanding of the clinicopathological features, prognostic factors and the oncogenic pathways, its identification and management can be a diagnostic challenge. This case report describes an asymptomatic complex liver cystic lesion in a 63-year-old patient, which proved to be cystic type IPNB.

The work has been reported in line with the Scare 2020 guidelines [4].

## 2 Presentation of case

A 63-year-old Caucasian man presented to our unit after a routine abdominal ultrasound which revealed a new multilocal cystic lesion in the left liver lobe. The patient has a previous medical history of metabolic syndrome with diabetes mellitus type 2, hypertension, hepatic steatosis, and chronic obstructive pulmonary disease.

**Fig. 1:**
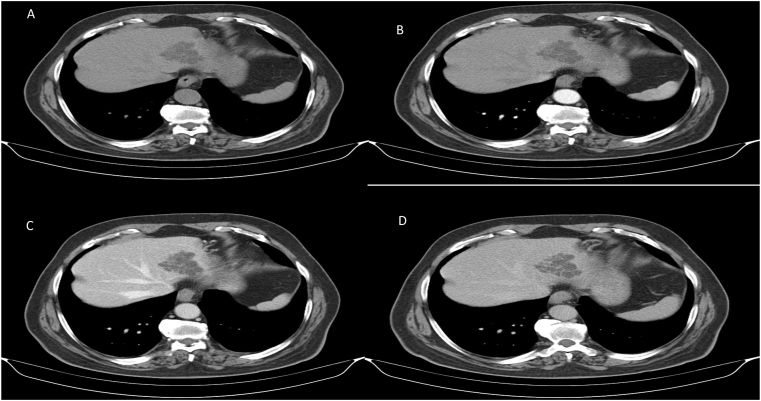
Image 1a: CT axial without contrast: a lobulated hypointense, cystic-type mass is depicted in liver segment ΙΙ, with 5 × 4cm dimensions. 1b: CT axial arterial phase shows the multilocular cystic mass with a few mild enhancing internal septa.1c: CT axial portal venous phase shows the well-defined multilocular intrahepatic cystic mass with multiple enhancing internal septa. There is no dilatation of the intrahepatic biliary ducts.1d: CT axial delayed phase shows the thick internal septa with persisted enhancement.

**Fig. 2:**
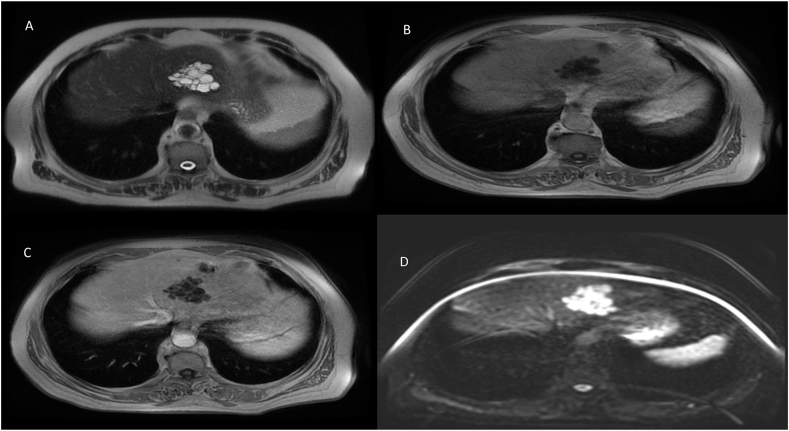
Image 2a: T2-weighted MRI axial: a well-defined multilocular intrahepatic mass is depicted, with high-intensity fluid content and multiple internal septa. 2b: T1-weighted unenhanced MRI axial: the lobulated intrahepatic mass has low heterogeneous signal intensity. 2c: T1-weighted MRI axial portal, delayed phase: the enhancement of the internal septa, is more obvious.2d: Diffusion-weighted MRI (ADC): the fluid in the cystic lesion show no restricted diffusion. There are thick internal septa.

The patient underwent a four-phase abdominal computed tomography (CT)) scan. On unenhanced CT scans, a lobulated hypointense, cystic-type mass was depicted in liver segment ΙΙ, and the dimensions were 4,7x4,5x3,3cm (Fig. 1a). On enhanced CT hepatic arterial and portal phase images, the lobulated contour of the multilocular intrahepatic cystic mass was well-defined, and multiple enhancing internal septa were observed. There was no dilatation of the intra- or extrahepatic biliary tree (Fig. 1b and c). On delayed phase images, the multiple internal septa showed continuous enhancement (Fig. 1d). No ascites in the abdominal cavity or enlarged lymph nodes were observed in the abdominal cavity or retroperitoneum. For further evaluation of the lesion, MRI was carried out subsequently. On T2-weighted MRI images, a well-defined multilocular, grape-like multicystic, intrahepatic mass was depicted in liver -segment II, with high signal intensity fluid content and multiple internal septa (Fig. 2a). Compared to normal hepatic parenchyma, the lesion showed a hypointense signal on unenhanced T1-weighted images (Fig. 2b). During the arterial, portal and delayed phases of dynamic enhanced MRI, the multiple internal septa of the lesion, showed moderate progressive enhancement (Fig. 2c). On diffusion-weighted images (DWI), the fluid in the multi-cystic lesion showed no restricted diffusion (Fig. 2d). In summary, the lesion had an intrahepatic peripheral location and showed a multilocular, grape-like multi-cystic appearance, with no obvious communication with the biliary tree, which showed a gradual progressive enhancement of the multiple internal septa. The radiologic features were not specific.

The liver function tests were normal. Tumours markers (CEA, CA 19-9, AFP) were within normal range, and anti– echinococcal antibodies were negative. Serology for hepatitis B and C infection was also negative. Based on suspected malignancy on imaging examinations, surgical resection was decided.

In theatre, a 5 cm complex cystic lesion was found in segments II/IVa. The patient underwent a left hepatectomy and cholecystectomy by a senior hepato-biliary Surgeon with more than 20 years of experience in liver surgery. The postoperative course was uneventful.

**Fig. 3:**
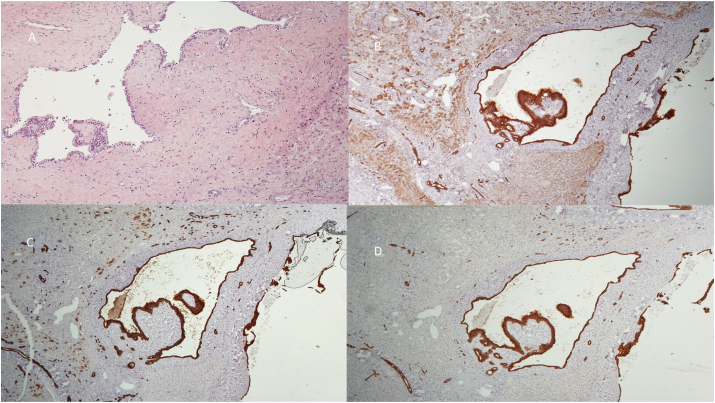
Within the liver parenchyma, there are cystic spaces lined by columnar or cuboidal epithelium, without cytologic atypia. Papillary projections with fibrovascular cores are noted. Fibrous tissue surrounds the cysts. A. Haematoxylin-eosin (X200), B. Immunostaining for cytokeratin 8 (DABX400), C. Immunostaining for cytokeratin 7 (DABX400), D. Immunostaining for cytokeratin 19 (DABX400).

Gross examination of the left hepatectomy specimen revealed a 4.7 × 4.2 cm complex cystic lesion. Microscopic examination showed multiple cysts lined by single-layered epithelial cells, mostly columnar with mucinous cytoplasm, but also cuboidal or flattened with eosinophilic cytoplasm. The nuclei were basally oriented, and there was no cellular atypia. Papillary projections, mostly with prominent fibrovascular cores, were noted protruding within the cystic spaces. Upon immunohistochemical examination, the epithelial lining cells were positive for cytokeratins 7, 8, 18, 19 and negative for ER, RR, chromogranin, synaptophysin. There was no endometrial-like stroma surrounding the cysts. The histological characteristics were consistent with a cystic type intrahepatic IPNB (Fig. 3). The hepatic resection margins were negative for tumour, with a clear margin over 8mm (R0 resection).

The patient is in good health without signs of recurrence 2 yrs after the operation.

## 3 Discussion

IPNB is a relatively new entity, was adopted in the 2010 World Health Organization classification as a precursor lesion of cholangiocarcinoma, deriving from the normal epithelium of the bile duct and progress through low-, intermediate-, and high-grade intraepithelial neoplasia to invasive carcinoma [5,6].

The highest incidence reported in Eastern countries, where hepatolithiasis [7] and clonorchiasis, which are believed to be major risk factor for IPNB are endemic. It is very rare in the West. Most patients are between 50 and 70 years old and male predominance is reported [2,7].

Pathogenesis is not clear. IPNB probably is caused by cholestasis and biliary tract infection. Furthermore, a multistage carcinogenesis to hyperplasia-dysplasia-carcinoma sequence is proposed as a mechanism for biliary tract cancer due to chronic inflammation [8].

Clinical features of IPNB usually relate to biliary obstruction secondary to tumour invasion or mucin production, and include right upper quadrant pain, jaundice, elevated liver enzymes, or recurrent cholangitis; however, some patients can be asymptomatic [[9], [10], [11]]. Approximately 30% of patients have a previous history or concomitant existence of biliary stones, as described in reports from Eastern countries [9].

There are four different radiological patterns of IPNB depending on the size and morphology of the intraductal mass, mucin secretion and neoplasm location. These are: intraductal mass with proximal and distal ductal dilatation, dilatation without mass, intraductal mass with proximal ductal dilation, which is the most common subtype and cystic-type lesion [11]. The most common imaging findings of IPNB include dilated bile ducts, intraluminal mucin and lesions protruding into the lumen. Multiple lesions can be find often (up to 50%) [11].

Abdominal ultrasound is the first imaging modality performed, where diffuse dilated bile duct without visible mass, focal dilated bile duct with intraductal papillary masses can be seen [12]. Contrast-enhanced ultrasonography (CEUS) can evaluate the malignancy potential. Hyperenhancement during the arterial phase of CEUS corresponds to malignant tissue, concluding that CEUS is an effective and meaningful imaging feature for analysing IPNB extension [12].

For further investigation CT scan is needed to detect intraductal masses, although its sensitivity is reported to be in the range of 50%. MRI and MRCP have the highest sensitivity [11].

Our case was a cystic type IPNB without communication with the biliary tree. Cystic type IPNB has similar imaging features with other cystic liver lesions such as mucinous cystic neoplasms, complicated hepatic cysts, localised Caroli disease [13]. A communication between the cystic type IPNB and bile duct, or bile duct dilatation can be a clue to differentiate it from other cystic lesions [11]. Hyperenhancement of the cystic wall or nodules in cystic type IPNB is an important finding for evaluating the potential for malignancy [13].

IPNB, a precursor lesion with good surgical prognosis, should be distinguished from cholangiocarcinoma. The triad of a local dilation of the bile duct, a nodule within the dilated duct, and a growth along the interior wall of the bile duct on CT or MRI has a positive predictive value above 90% for identifying IPNB vs cholangiocarcinoma [[13], [14]].

According to WHO, IPNB is to be classified by its pathology into 4 epithelial subtypes: pancreatobiliary, intestinal, gastric, and oncocytic, with mucin secretion either present or absent.

They are further classified into 3 types regarding location: intrahepatic, extrahepatic and diffuse type. Intrahepatic is the most common type. Extrahepatic- and diffuse-type IPNB tended to be more aggressive than intrahepatic type [15]. Recently branch type IPNB was reported [16,17]. Small brunch type intrahepatic IPNB often look like simple liver cysts making the diagnosis of IPNB difficult.

The primary treatment decision for IPNB is surgical resection to alleviate biliary obstruction and treat or prevent malignancy. Surgical resection is considered for patients without distant metastasis and includes major hepatectomy with or without extrahepatic bile duct resection. Intraoperative frozen section of the bile duct stump should be performed to ensure a negative margin, given the tendency of these tumours to spread superficially along the bile duct and their multifocal nature [18,19].

The application of laparoscopic hepatectomy for small tumours has been reported [17]. Also, there are case reports where follow up was suggested for small lesions [11].

IPNB has a better prognosis and postsurgical outcomes than conventional intraductal neoplasia-associated cholangiocarcinoma. In a large multicenter cohort study from South Korea, 387 patients with histologically proven IPNB were studied. Almost half of them (45,5%) had invasive carcinoma. The 5-year overall survival was 80,9% for all patients, 89% for IPNB with mucosal dysplasia and 70,5% for IPNB with invasive carcinoma. The majority of the lesions were intrahepatic (69%). Multivariate analysis showed that tumour invasiveness was a significant predictor for survival [20]. Major prognostic post-surgery factors include tumour invasiveness, resection margin status, tumour multiplicity and, lately, tumour location. The recurrence rate has been reported to be high even after a long period since surgical resection, due to preoperatively undetected remote lesions [21]. Thus, long-term follow-up is needed even after complete curative resection of IPNB.

## 4 Conclusion

We report a case of a Caucasian male without prior medical history related to the biliary tree and the liver; presented with an asymptomatic cystic type IPNB. He underwent left hepatectomy and cholecystectomy. Histological examination of the tumour shows prominent papillary proliferation and delicate fibrovascular cores lacking ovarian type stroma. Notably, two years later, the patient remains disease-free.

Taken together, IPNB is a rare bile duct epithelial tumour with a very low incidence in the West and constitutes a precursor to cholangiocarcinoma development. It is important to emphasise that although IPNB is a potentially curable disease, it requires R0 resection and even then, a close follow-up is recommended.

## Sources of funding

There was no funding.

## Ethical approval

Ethics committee approval has been given.

## Consent

Written full informed consent was obtained by the patient.

## Author contribution

Evangelos G Baltagiannis, Christina Kalyvioti: Data collection and writing the paper. Anastasia Glantzouni: Radiological data interpretation and writing the relative chapter. Prof. Anna Batistatou: Histopathological data interpretation and writing the relative chapter. Prof. Petros Tzimas: critical revision of the manuscript. Prof. Georgios K. Glantzounis: Study concept, manuscript revision.

## Registration of Research Studies

Name of the registry: non applicable.

Unique Identifying number or registration ID: non applicable.

Hyperlink to your specific registration (must be publicly accessible and will be checked): non applicable.

## Guarantor

Georgios K. Glantzounis, MD, PhD, FEBS.

Professor of Surgery and Transplantation.

Head of HPB Unit.

Department of Surgery, School of Medicine.

University of Ioannina, 45 110.

Ioannina, Greece.

Tel: 00302651099695, 00306984189292.

Fax: 00302651099890.

E-mail: gglantzounis@uoi.gr; gglantzounis@gmail.com

## Provenance and peer review

Not commissioned, externally peer-reviewed.

## Declaration of competing interest

There are no conflicts of interest.

## References

[1] Sato T., Hisaka T., Sakai H. (2019). Clinicopathological study of resections of intraductal papillary neoplasm of the bile duct. Anticancer Res..

[2] Onoe S., Shimoyama Y., Ebata T. (2014). Prognostic delineation of papillary cholangiocarcinoma based on the invasive proportion: a single-institution study with 184 patients. Surgery.

[3] Fukumura Y., Nakanuma Y., Kakuda Y., Takase M., Yao T. (2017). Clinicopathological features of intraductal papillary neoplasms of the bile duct: a comparison with intraductal papillary mucinous neoplasm of the pancreas with reference to subtypes. Virchows Arch..

[4] Agha R.A., Franchi T., Sohrabi C. (2020). The SCARE 2020 guideline: updating consensus surgical CAse REport (SCARE) guidelines. Int. J. Surg..

[5] Nakamura Y., Curabo M.O., Franceschi S. (2010). Intrahepatic Cholangiocarcinoma. WHO Classification of Tumors of the Digestive System. World Health Organization Tumors.

[6] WHO Classification of Tumours (2019).

[7] Wang X. (2015). Biliary tract intraductal papillary mucinous neoplasm: report of 19 cases. World J. Gastroenterol..

[8] Aishima S., Kubo Y., Tanaka Y., Oda Y. (2014). Histological features of precancerous and early cancerous lesions of biliary tract carcinoma. J. Hepatobiliary. Pancreat. Sci..

[9] Ohtsuka M., Kimura F., Shimizu H. (2011). Similarities and differences between intraductal papillary tumors of the bile duct with and without macroscopically visible mucin secretion. Am. J. Surg. Pathol..

[10] Kim K.M., Lee J.K., Shin J.U. (2012). Clinicopathologic features of intraductal papillary neoplasm of the bile duct according to histologic subtype. Am. J. Gastroenterol..

[11] Park H.J., Kim S.Y., Kim H.J. (2018). Intraductal papillary neoplasm of the bile duct: clinical, imaging and pathologic features. Am. J. Roentgenol..

[12] Liu L.N., Xu H.X., Zheng S.G. (2015). Ultrasound findings of intraductal papillary neoplasm in bile duct and the added value of contrast-enhanced ultrasound. Ultraschall der Med..

[13] Hasebe T., Sawada K., Hayashi H. (2019). long-term growth of intrahepatic papillary neoplasms: a case report. World J. Gastroenterol..

[14] Liu Y., Zhong X., Yan L., Zheng J., Liu Z., Liang C. (2015). Diagnostic performance of CT and MRI in distinguishing intraductal papillary neoplasm of the bile duct from cholangiocarcinoma with intraductal papillary growth. Eur. Radiol..

[15] Kim J.R., Lee K.B., Kwon W., Kim E., Kim S.W., Jang J.Y. (2018). Comparison of the clinicopathologic characteristics of intraductal papillary neoplasm of the bile duct according to morphological and anatomical classifications. J. Kor. Med. Sci..

[16] Nakamura Y., Kakuda K., Uesaka K. (2016). Characterisation of intraductal papillary neoplasm of the bile duct respect to histopathologic similarities to pancreatic intraductal papillary mucinous neoplasm. Hum. Pathol..

[17] Matono R., Nonomiya M., Morita K. (2020). Branch-type intraductal papillary neoplasm of the bile duct treated with laparoscopic anatomical resection: a case report. Surg. Case Rep..

[18] Zhang X.F., Squires M.H., Bagante F. (2018). The impact of intraoperative re-resection of a positive bile duct margin on clinical outcomes for hilar cholangiocarcinoma. Ann. Surg Oncol..

[19] Tominaga K., Kamimura K., Sakamaki A., Terai S. (2017). Intraductal papillary neoplasm of the bile duct: a rare liver tumor complicated by malignancy. Hepatology.

[20] Kim J.R., Jang K.T., Jang J.Y. (2020). Clinicopathological analysis of intraductal papillary neoplasm of bile duct; Korean multicentre cohort study. HPB.

[21] You Y., Choi S.H., Choi D.W. (2020). Recurrence after resection for intraductal papillary neoplasm of bile duct (IPNB) according to tumor location. J. Gastrointest. Surg..

